# Draft genome of the *Ogataea polymorpha* type strain CBS 4732

**DOI:** 10.1128/MRA.00611-22

**Published:** 2023-08-02

**Authors:** Joseph H. Collins, Eric M. Young

**Affiliations:** 1 Department of Chemical Engineering, Worcester Polytechnic Institute, Worcester, Massachusetts, USA; Vanderbilt University, Nashville, Tennessee, USA

**Keywords:** yeast genomics, nanopore sequencing

## Abstract

The methylotrophic yeast *Ogataea polymorpha* is of significant biotechnological interest, particularly in high-temperature fermentations and for recombinant protein production. Here, we present a high-quality genome assembly for the *O. polymorpha* type strain CBS 4732.

## ANNOUNCEMENT

*Ogataea polymorpha*, formerly *Hansenula polymorpha*, is thermotolerant, methylotrophic, catabolizes various carbon sources, and produces proteins with desirable glycosylation patterns. Thus, it is an attractive host for therapeutics (1) and conversion of biomass to fuels and chemicals (1, 2). Genetic tools are available (3), and engineering efforts have been successful in generating improved strains (4, 5). Here, we report the draft genome of the type strain *O. polymorpha* CBS 4732. This genome is larger and more complete than two other publicly available *O. polymorpha* genomes, NCYC 495 leu1.1 (6) and BY4329 (7), shown in Table 1. It is also more complete than strain RB11, an engineered *odc1* derivative of CBS 4732, which is not publicly available (8). This improved public genome assembly will aid in studying *O. polymorpha* genetics and the design and validation of engineered strains.

**TABLE 1:** Genome structure and completeness comparison of available *O. polymorpha* strains^*a*^

Strain	Size (Mb)	Contigs	N50 (Mb)	*N*s per 100 kbp	BUSCO score
NCYC 495 leu1.1	8.97	7^*b*^	1.30	0.04	94.57
BY4329	8.97	17	1.30	836.12	94.48
CBS 4732	9.02	8	1.32	0.00	94.62

^*a*^ *O. polymorpha* NCYC 495 leu1.1 was obtained from Riley et al. (6), *O. polymorpha* BY4329 was obtained from Maekawa et al. (7), and *O. polymorpha* CBS4732 is from this work.

^*b*^ Does not include mitochondrion.

*O. polymorpha* CBS 4732 was purchased from the American Type Culture Collection (ATCC 34438), revived, and cryogenically stored according to the product sheet. It was cultivated from the cryostock in 5 mL of yeast extract peptone dextrose (YEPD), 10 g/L yeast extract, 20 g/L peptone, and 20 g/L glucose) for 2 d at 30°C. Genomic DNA isolation and sequencing followed protocols previously published (9). Briefly, genomic DNA was isolated using Promega’s Genomic DNA Isolation Kit (Promega, A1120). The nanopore DNA library was prepared using the Rapid Barcoding Kit (Oxford Nanopore Technologies, SQK-RBK004) and sequenced on the Oxford Nanopore Technologies MinION. The Illumina DNA library was prepared using the Nextera DNA Flex Library Prep Kit (Illumina, 20018704) along with the Nextera DNA CD Indexes (Illumina, 20018707), and sequencing was conducted on the Illumina iSeq 100.

For all following software steps, default parameters were used unless otherwise noted. Nanopore fast5 files were basecalled using Guppy v2.3.5 (Oxford Nanopore base caller). The subsequent fastq files were demultiplexed using the EPI2ME interface (Metrichor, Oxford, UK). Illumina reads were demultiplexed using the native software on the iSeq 100 machine. A total of 120,609 nanopore reads with an average read length of 4,876 bp (63× genome coverage) and 931,187 paired-end Illumina reads with an average read length of 148 bp (31× genome coverage) resulted from basecalling and demultiplexing, which also filtered out low-quality reads according to default parameters.

Genome assembly and polishing were done with Prymetime v0.2 hybrid assembly software (9). Structural statistics (genome size, GC content, number of contigs, N50, and assembly gaps) were calculated using QUAST v4.6.3 (10), while genome completeness was assessed using BUSCO v4.0.6 with the Saccharomycetes database (11). The *O. polymorpha* CBS 4732 assembly had a GC content of 47.65% with a total size of 9.02 Mb. This assembly compared well to the other *O. polymorpha* assemblies (Table 1).

## Data Availability

Data were deposited at GenBank under BioProject number PRJNA655503 (BioSample number SAMN15738545, SRA numbers SRR12403033 and SRR12403034, and assembly number GCA_017161175.1).
